# Manganese (II) chloride leads to dopaminergic neurotoxicity by promoting mitophagy through BNIP3-mediated oxidative stress in SH-SY5Y cells

**DOI:** 10.1186/s11658-021-00267-8

**Published:** 2021-06-02

**Authors:** Yanning Huang, Qiaolin Wen, Jinfeng Huang, Man Luo, Yousheng Xiao, Ruikang Mo, Jin Wang

**Affiliations:** 1 Department of Neurology, The First Affiliated Hospital of Guangxi Medical University, Nanning, 530021 China; 2 Department of Neurology, Liuzhou Worker’s Hospital, Liuzhou, 545005 China; 3 Department of Neurology, First Peoples Hospital of Nanning, Nanning, 530021 China

**Keywords:** Manganese, Neurotoxicity, Mitophagy, BNIP3, Oxidative stress

## Abstract

**Background:**

Manganese overexposure can induce neurotoxicity, lead to manganism and result in clinical manifestations similar to those of parkinsonism. However, the underlying molecular mechanism is still unclear. This study demonstrated that MnCl_2_ induces mitophagy and leads to neurotoxicity by promoting BNIP3-mediated reactive oxygen species (ROS) generation.

**Methods:**

Human neuroblastoma SH-SY5Y cells were used throughout our experiments. Cell viability was detected by cell proliferation/toxicity test kits. Mitochondrial membrane potential was measured by flow cytometry. ROS generation was detected using a microplate reader. Protein levels were evaluated by Western blot. Transmission electron microscopy was used to evaluate mitochondrial morphology. Co-immunoprecipitation was used to verify the interaction between BNIP3 and LC3.

**Results:**

MnCl_2_ led to loss of mitochondrial membrane potential and apoptosis of SH-SY5Y cells by enhancing expression of BNIP3 and conversion of LC3-I to LC3-II. Moreover, MnCl_2_ reduced expression of the mitochondrial marker protein TOMM20 and promoted interaction between BNIP3 and LC3. The results also indicated that a decrease in BNIP3 expression reduced the mitochondrial membrane potential loss, attenuated apoptosis and reduced mitochondrial autophagosome formation in SH-SY5Y cells after MnCl_2_ treatment. Finally, we found that manganese-induced ROS generation could be reversed by the antioxidant N-acetyl cysteine (NAC) or silencing BNIP3 expression.

**Conclusions:**

BNIP3 mediates MnCl_2_-induced mitophagy and neurotoxicity in dopaminergic SH-SY5Y cells through ROS. Thus, BNIP3 contributes to manganese-induced neurotoxicity by functioning as a mitophagy receptor protein.

## Background

Parkinsonism is a neurological disease characterized by dyskinesias such as tremor, rigidity, akinesia, and postural disturbances. In recent years, the overall incidence of Parkinsonism has ranged from 23 to 59 (per 100,000 persons) in women, and from 25 to 75 in men, varying among different investigations [1–3]. Dopamine levels gradually decrease with nigrostriatal dopamine degeneration or dysfunction. The deposition of alpha-synuclein and tau protein is considered to be the critical pathological characteristic of parkinsonism [4]. It is generally agreed that the incidence of parkinsonism increases with aging and the presence of genetic factors and various environmental factors, including pesticides, insecticides, metals and other industrial chemicals [5, 6]. Some metals, such as iron, manganese, and copper, are present in our daily diet. At low concentrations and under certain conditions, these metals participate in various biological functions in the body. However, in overexposure situations, such as in industrial and commercial applications, metal concentrations exceeding the physiological concentrations required in the body have serious adverse effects, especially on the nervous system. When metals accumulate in the brain, they can migrate to different regions of the brain and interact with different neurons, stimulating cells to produce highly reactive substances, such as reactive oxygen species (ROS) and reactive nitrogen species (RNS), which affect related genes and their associated regulatory pathways in neurons, leading to cell and organ dysfunction [7]. Occupational or environmental pollution exposure can result in excessive manganese exposure, which makes the relationship between neurotoxic effects and parkinsonism-like symptoms caused by manganese a concern. At physiological concentrations, manganese not only participates in many biological processes as a coenzyme but also mediates the synthesis and metabolism of neurotransmitters [8], but excessive accumulation of manganese can adversely affect the body. Excessive manganese accumulation in the brain can lead to clinical manifestations similar to those of parkinsonism, and this condition is known as manganism [9]. Studies have shown that excessive exposure to manganese can affect multiple cellular processes, such as ROS generation [10], mitochondrial dysfunction [11], and autophagy [12], and then lead to neurotoxicity [13]. In recent years, the emergence of molecular imaging has led to further understanding of manganism [14]. Although research on manganese exposure and its relevance to parkinsonism has increased annually, the underlying molecular mechanism is still unclear.

Mitochondria, the primary source of cellular ROS, are the intersection of various biological regulatory processes and are closely related to human health. In many neurodegenerative diseases, ROS generated by the damaged mitochondria often cause harmful oxidative stress to cells, and thus contribute to disease progression [15]. Mitophagy is an effective way to reduce oxidative damage, which includes mitochondrial autophagic degradation and the formation of new mitochondria [16]. Mitophagy is particularly important for preventing cell damage by maintaining mitochondrial homeostasis. Currently, a well-known mitophagy-mitochondrial fusion/fission dynamic equilibrium system is the p62/SQSTM1/NBR1 pathway and the ubiquitination regulation pathway of PINK1/parkin [17, 18]. In recent years, some mitophagy receptor proteins, such as BNIP3 [19, 20], BNIP3 homolog BNIP3L [21] and FUNDC1 [22], which have specific transmembrane domains and are localized to the outer mitochondrial membrane (OMM) [23, 24], have been shown to regulate mitophagy and mitochondrial fusion/splitting homeostasis by interacting with the autophagy marker protein LC3 through the LC3-interacting region motif. As a member of the Bcl2 family, BNIP3 can inhibit anti-apoptotic proteins such as E1B19 kDa and Bcl2 and promote apoptosis. When mitochondria are damaged, BNIP3 can participate in mitochondrial protein catabolism and promote the degradation of damaged proteins in mitochondria through mitochondrial quality control. In addition, after BNIP3 is activated, it can regulate the opening of mitochondrial membrane permeability transition pores to mediate lysosomal protein transfer from the cytoplasm to the mitochondrial matrix and the biological regulation of mitochondrial dysfunction [25]. BNIP3 has been found to be a target regulatory gene for manganese-induced neurotoxicity, which is associated with mitochondrial dysfunction and oxidative stress [26]. The purpose of this study was to investigate the BNIP3-dependent neurotoxic effects of MnCl_2_ on human neuroblastoma SH-SY5Y cells. To this end, we used the dopaminergic cell line SH-SY5Y as a cell model and MnCl_2_ as a stimulus to explore the regulation of BNIP3-mediated, MnCl_2_-induced mitophagy in dopaminergic neurons. Hopefully, this work can provide new insights into the pathophysiological mechanisms of parkinsonism.

## Methods

### Reagents

Manganese (II) chloride tetrahydrate (MnCl_2_) and sodium dodecyl sulfate-polyacrylamide gel (SDS-PAGE) were purchased from Sigma-Aldrich (St. Louis, MO, USA). Fetal bovine serum (FBS) was obtained from Gibco (Grand Island, NY, USA). Dulbecco’s modified Eagle’s medium (DMEM) was purchased from Corning (Tewksbury, MA, USA). Penicillin streptomycin, trypsin-EDTA solution, phosphate buffer saline (PBS), sodium dodecyl sulfate (SDS), ammonium persulfate (AP), and tris (hydroxymethyl) aminomethane (Tris) were obtained from Beijing Suobao Science & Technology Co., Ltd. (Beijing, China). BNIP3 short hairpin (shRNA) lentivirus, negative control lentivirus, and polybrene were obtained from Shanghai Jikai Gene Chemical Technology Co., Ltd. (Shanghai, China). Puromycin, the mitochondrial membrane potential detection kit with JC-1 (JC-1), and the BCA protein assay kit were purchased from Beyotime Biotech Inc (Shanghai, China). The cell proliferation/toxicity test kit CCK-8 was purchased from Dojindo Laboratories. (Shanghai, China). Anti-BNIP3 antibody and anti-TOMM20 antibody were purchased from Abcam (Cambridge, MA, USA). Anti-LC3B antibody was purchased from Novus Biologicals (Littleton, CO, USA). Anti-β-actin antibody was purchased from Cell Signaling Technology (Danvers, MA, USA). The secondary antibody was purchased from LI-COR Biosciences Inc. (Lincoln, NE, USA). Testing equipment: the enzyme labeling instrument was produced by BioTek (BioTek, Winooski, Vermont, USA), the flow cytometer was produced by Becton Dickinson and Company (Franklin Lakes, NJ, USA), the electrophoresis instrument was produced by Bio-Rad (Hercules, CA, USA), and the Odyssey two-color infrared laser imaging system was produced by LI-COR Biosciences Inc. The electron microscope was produced by Hitachi (Tokyo, Japan).

### Cell culture

SH-SY5Y human neuroblastoma cells were obtained from American Type Culture Collection (ATCC, Manassas, VA, USA). Cells were maintained in DMEM containing 10 % FBS and 1 % penicillin/streptomycin and incubated at 37 °C in 95 %/5 % CO_2_ air. Cells were grown in 25 cm^2^ culture flasks to 80 % confluence. The study was approved by the Ethics Committee of the First Affiliated Hospital of Guangxi Medical University.

### Cell viability assay

SH-SY5Y cell inhibition by MnCl_2_ was measured by the CCK-8 assay. Cells (1 × 10^4^ cells/mL) were placed in 96-well plates and treated with 0, 200, 400, 800, or 1600 µM MnCl_2_ for 12, 24 and 48 h in the incubator. After intervention with the various concentrations of MnCl_2_, 10 µL of CCK-8 solution was added to each well, and cells were incubated for 2 h at 37 °C. The absorbance was determined using a microplate reader at 450 nm (BioTek ELX800, USA).

### Lentivirus vector and cell transfection

SH-SY5Y cells were transduced with LV-BNIP3-RNAi lentivirus carrying shRNA designed to knock down BNIP3 and cloned into the GV112 vector. The virus titer was 5E + 8 TU/mL. GV115-NC lentivirus with no interference sequence was used to infect SH-SY5Y cells as a negative control. The virus titer was 5E + 8 TU/mL. Cells were cultured overnight in 6-well plates, and the constructed vector was transfected into SH-SY5Y cells using 2 mL of DMEM with 5 µg/mL of polybrene. After 48 h, the medium was replaced with fresh medium, and the transduced cells were positively selected by continuous exposure to 1 µg/mL puromycin for 15 days. After selection, the knockdown efficiency of BNIP3 was determined by western blot analysis.

### ROS generation evaluation

Cells were cultured for 24 h after treatment with 400 µM MnCl_2_. After treatment, cells were incubated with 10 µM 2′,7′-dichlorodihydrofluorescein diacetate (DCFH-DA, Sigma) in serum-free cell culture medium at 37℃ for 20 min. Then, DCFH-DA fluorescence was monitored using a microplate reader (BioTek Synergy H1, Switzerland). Here, 525 nm was the emission wavelength, and 488 nm was the excitation wavelength.

### Mitochondrial membrane potential and apoptosis assays

Cell density was adjusted to approximately 20 × 10^4^/mL, and cells were incubated at 37 °C for 30 min in the presence of JC-1 through the use of a mitochondrial membrane potential assay kit (1×, 2 µM, Beyotime, China). Then, the cells were washed with cold JC-1 buffer 3 times for 15 min, and the fluorescence intensity of the cells was analyzed by flow cytometry (BD FACSCalibur, USA). A total of 2 × 10^5^ cells were collected. To each tube, 400 µL of 1X binding buffer was added. Then, 5 µL of fluorescein isothiocyanate (FITC) Annexin V and 5 µL of PI were added. The cells were gently vortexed and incubated for 15 min in the dark. The apoptosis rate of cells was analyzed by flow cytometry (BD FACSCalibur, USA).

### Western blot analysis

Cells were treated with MnCl_2_ and/or other reagents. After the medium was discarded, the cells were rinsed twice with PBS, scraped and collected on ice. Then, RIPA and PMSF (100:1) were added, followed by cryopreservative, and the sample was centrifuged (12 000 rpm for 15 min at 4 °C); the supernatant contained the total protein extract. Protein concentrations were determined by the BCA protein assay kit. Then, 30 µg of protein was added to each lane of a gel, and the ionophores were separated by 12–15% SDS-PAGE. The cells were transferred to an Immobilon-P Transfer membrane (Millipore, USA). After blocking with 5 % skim milk for 1 h, membranes were incubated with anti-LC3 (1:1000, NB600-138SS, Novus), anti-BNIP3 (1:1000, ab109362, Abcam), anti-TOMM20 (1:1000, ab186735, Abcam), and anti-β-actin (1:1000, #8457, CST) overnight at 4 °C. Then, the membranes were washed 3 times with TBST (1×) for 15 min and incubated with secondary antibody at room temperature for 1 h (1:10,000, C40723-01, LI-COR). Protein signals were detected by an Odyssey two-color infrared laser imaging system (LI-COR).

### Coimmunoprecipitation

After the cells reached 70–80 % confluence, the medium was discarded, and the cells were washed twice with ice-cold PBS and lysed on ice for 30 min. Then, the cells were scraped off with a cell scraper and collected into a 1.5-mL centrifuge tube. The cells were centrifuged at 12 000 rpm for 30 min at 4 °C. After centrifugation, the supernatant was carefully aspirated, and the supernatant contained the total protein extract. After the total protein concentration was determined by the BCA protein assay kit, the appropriate amount of protein was incubated with anti-LC3 or anti-BNIP3. In the meantime, 5 µL of protein A agarose beads was washed with PBS twice, dissolved to 50 % concentration in PBS and stored at 4 °C. Pretreated protein A agarose beads (50 %) were added to 100 µL of the total protein extract, which had been incubated with anti-LC3 or anti-BNIP3 overnight with gentle shaking at 4 °C. After incubation, the cells were centrifuged at 12,000 rpm for 30 min at 4 °C, and the agarose beads were centrifuged to the bottom of the tube. The supernatant was removed, and the agarose beads were washed 4 times with 500 µL of ice-cold PBS. Appropriate 5× loading buffer was added to mix the agarose bead-antigen antibody complex, boiled for 5 min, and centrifuged at 12,000 rpm for 5 min at 4 °C. The supernatant was stored at -80 °C for later protein electrophoresis. The protein electrophoresis procedure was the same as that for western blot analysis.

### Transmission electron microscopy

SH-SY5Y cells (1 × 10^5^/ml) were seeded in 6-well dishes 24 h before the experiment. After treatment with 400 µM MnCl_2_ for 24 h, cells were collected and fixed with 3 % glutaraldehyde at 4 °C for 3 h. Then, the cells were washed three times with ice-cold PBS for 15 min and fixed with 1 % citric acid for 3 h. After fixation, dehydration, infiltration, embedding, polymerization, ultrathin sectioning and lead citrate staining were carried out, followed by gradient alcohol dehydration. After being dried, the mitochondrial autophagosome was observed by a Hitachi H7650 electron microscope (Hitachi H7650, Japan).

### Statistical analysis

Data were analyzed using GraphPad Prism6 software (La Jolla, CA, USA) to perform one-way analysis of variance (ANOVA). The difference between two groups was determined using an independent-sample T test. Statistical results are expressed as the mean ± standard deviation (S.D.). *P* < 0.05 implies a significant difference, and *P* < 0.01 implies a highly significant difference.

## Results

### Neurotoxic effects of MnCl_2_ on human neuroblastoma SH-SY5Y cells

**Fig. 1:**
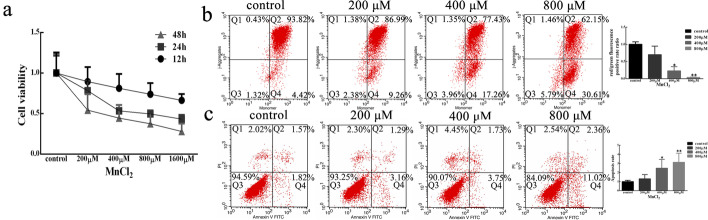
MnCl_2_ inhibited cell proliferation and induced apoptosis in SH-SY5Y cells. **a** SH-SY5Y cells were treated with 0–1600 μM MnCl_2_ for 12, 24, 48 h. Then, cell viability was measured by CCK-8 assays. **b**, **c** Mitochondrial membrane potential (**b**) and cell apoptosis (**c**) were observed using the flow cytometry method (FCM) after SH-SY5Y cells were treated with 200, 400, and 800 μM MnCl_2_

The purpose of this study was to investigate the neurotoxic effects of MnCl_2_ on human neuroblastoma SH-SY5Y cells. After treatment with different concentrations of MnCl_2_ for different time periods, the cell proliferation/toxicity test kit CCK-8 was used to determine the cell viability of SH-SY5Y cells. After 12 h, the inhibition of SH-SY5Y cells treated with different concentrations of MnCl_2_ was not significantly different from that of cells treated with the control treatment. However, after 24 and 48 h, the activity of SH-SY5Y cells decreased significantly with different concentrations of MnCl_2_ (Fig. 1a); the IC_50_ values for 24 and 48 h were 585 ± 139 µmol/L and 345 ± 273 µmol/L, respectively. Therefore, except for special cases, subsequent experiments were carried out at 0, 200, 400, of 800 µM MnCl_2_ for 24 h. In addition, to further understand the toxic effects of MnCl_2_ on SH-SY5Y cells, we detected markers of early apoptosis and mitochondrial membrane potential using a JC-1 kit. The results showed that the mitochondrial membrane potential decreased in a concentration-dependent manner after treatment with 200, 400, and 800 µM MnCl_2_ for 24 h (Fig. 1b). In addition, the apoptosis rate was also determined by the FITC Annexin V Apoptosis Detection Kit I. As shown in Fig. 1c, MnCl_2_ induced SH-SY5Y cell apoptosis in a dose-dependent manner. The above data indicated that MnCl_2_ can decrease the activity of SH-SY5Y cells and cause neurotoxicity.

### MnCl_2_ induced mitophagy in SH-SY5Y cells

**Fig. 2:**
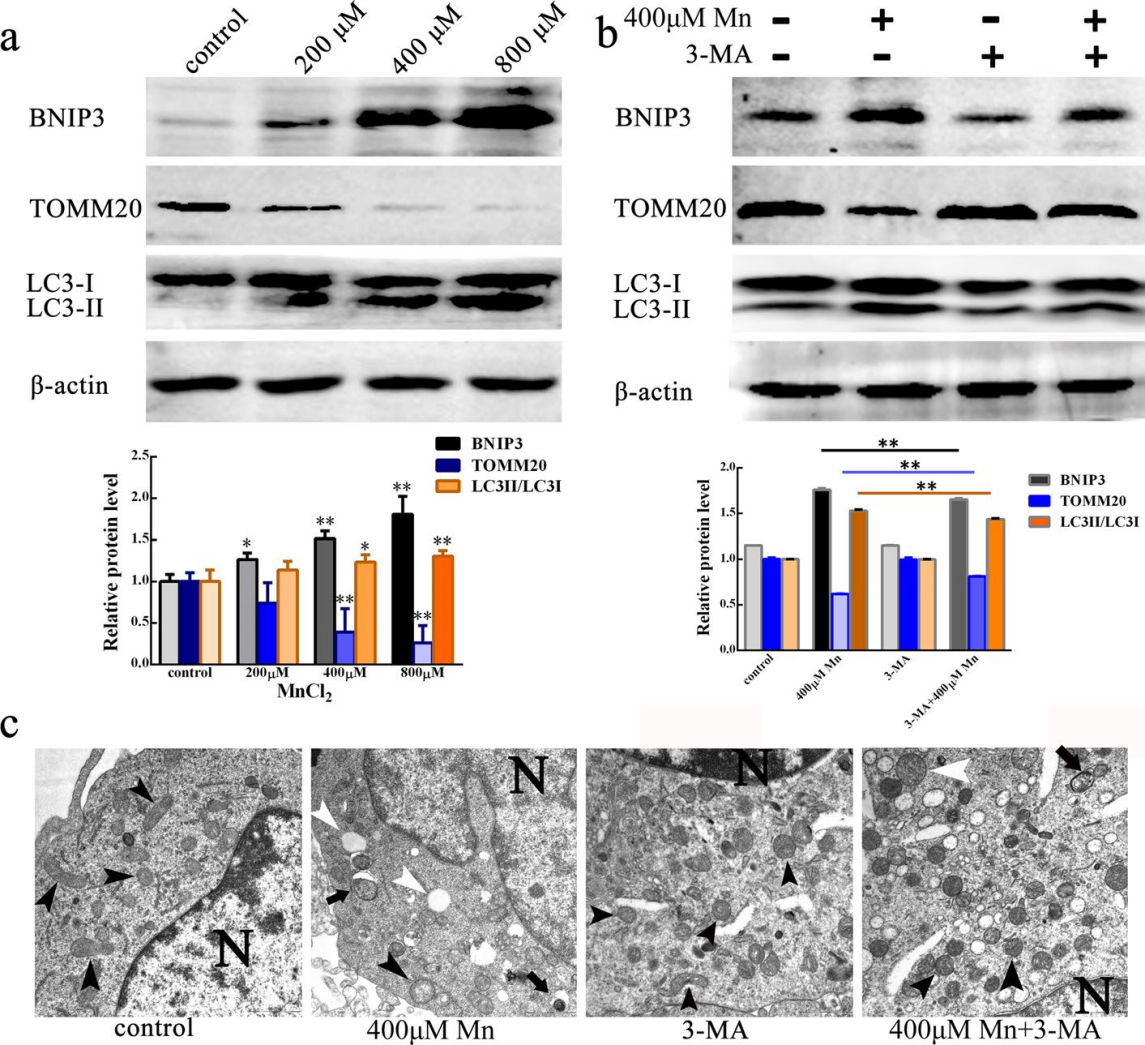
MnCl_2_ induced mitophagy in SH-SY5Y cells. **a**, **b** BNIP3, LC3 and TOMM20 protein levels were measured by western blot after SH-SY5Y cells were treated with 200, 400, 800 µM MnCl_2_ (**a**) and treated with 400 µM MnCl_2_ alone or co-treated with mitophagy inhibitor 3-MA together (**b**) for 24 h. β-actin served as an internal reference. Compared with control, *P < 0.05, **P < 0.01. **c** Mitophagy bodies and mitochondria treated with 400 μM MnCl2 alone or co-treated with mitophagy inhibitor 3-MA together for 24 h were observed by transmission electron microscopy (TEM). N, nucleus. White arrow, expanded, condensed or dissolved, mitochondria aggregate. Black arrowhead (  ), relatively normal mitochondrial morphology. Black arrow (  ), mitophagy bodies

To verify whether MnCl_2_ induced mitophagy in SH-SY5Y cells, we first evaluated the expression of the mitophagy receptor protein BNIP3 and the mitochondrial marker protein TOMM20 and the transformation of LC3-I to LC3-II by western blot analysis. LC3-II is a key marker in the process of mitophagy. As shown in Fig. 2a, compared with the control treatment, treatment with 200, 400 and 800 µM MnCl_2_ affected the expression of the mitophagy receptor protein BNIP3 and the conversion of LC3-I to LC3-II in a concentration-dependent manner, while the expression level of the mitochondrial marker protein TOMM20 decreased with increasing MnCl_2_ concentration. Next, we blocked mitophagy by using the autophagy inhibitor 3-MA to determine whether MnCl_2_ can induce mitophagy in SH-SY5Y cells. The results showed that 3-MA could reduce the expression of the mitophagy receptor protein BNIP3 and the conversion of LC3-I to LC3-II while increasing the expression of the mitochondrial marker protein TOMM20 after treatment with 400 µM MnCl_2_ for 24 h (Fig. 2b). Therefore, we inferred that MnCl_2_ induced mitophagy in SH-SY5Y cells. To directly and intuitively observe the formation of mitophagy bodies, we used transmission electron microscopy to evaluate mitochondrial morphological changes and mitophagic bodies in SH-SY5Y cells after treatment with 400 µM MnCl_2_ for 24 h. The results showed that, compared with the control treatment, treatment with 400 µM MnCl_2_ for 24 h resulted in expanded, dissolved, coagulated, or denatured mitochondria and induced the formation of mitochondrial autophagosomes with a bilayer membrane structure. Furthermore, 3-MA could alleviate MnCl_2_-induced mitochondrial autophagosome formation in SH-SY5Y cells (Fig. 2c). These data indicated that the formation of mitochondrial autophagosomes in SH-SY5Y cells was increased by MnCl_2_ exposure. The above data indicated that MnCl_2_ induced mitophagy in SH-SY5Y cells.

### ROS involvement in MnCl_2_-induced mitophagy in SH-SY5Y cells

**Fig. 3:**
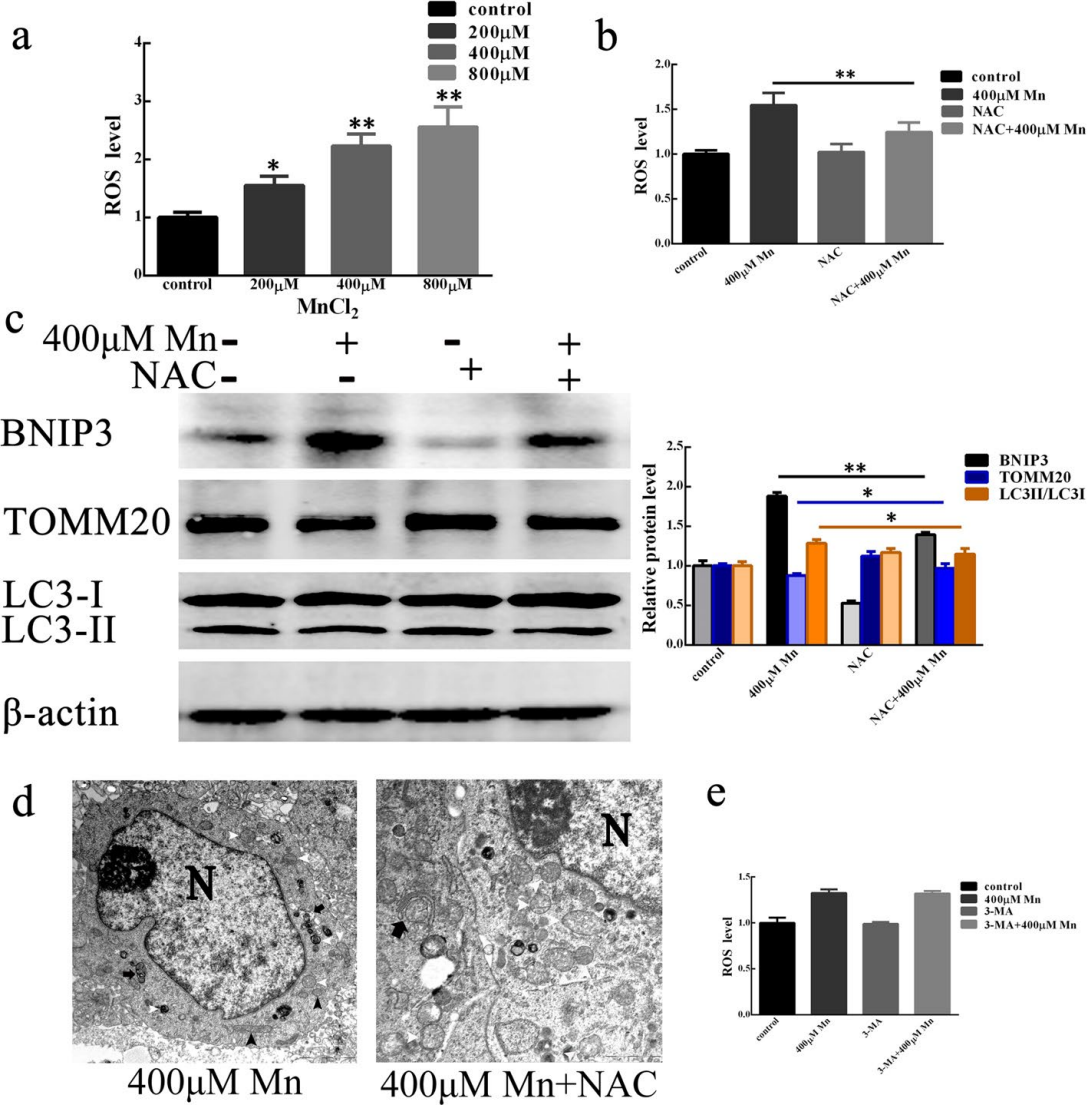
MnCl_2_ induced mitophagy by regulating ROS generation.  **a**, **b** SH-SY5Y cells were treated with 200, 400, and 800 µM MnCl_2_ (**a**) and 400 µM MnCl_2_ with or without pretreatment with the antioxidant NAC (**b**) for 24 h. ROS levels were measured using a microplate reader. **c** BNIP3, LC3 and TOMM20 protein levels were measured by western blot after treatment with 400 µM MnCl_2_ with or without pretreatment with the antioxidant NAC for 24 h. β-actin served as the internal reference. Compared with control, *P < 0.05, **P < 0.01. **d** Mitophagy bodies and mitochondria were observed by transmission electron microscopy (TEM) in cells treated with 400 μM MnCl2 alone or co-treated with the mitophagy inhibitor NAC for 24 h. N, nucleus. White arrow, expanded, condensed or dissolved, mitochondria aggregates. Black arrow (  ), relatively normal mitochondrial morphology. Black arrow (  ), mitophagy bodies. **e** After co-treatment with or without the autophagy inhibitor 3-MA, ROS levels in SH-SY5Y cells were also measured using a microplate reader

ROS are mainly produced in mitochondria. ROS may cause oxidative damage to mitochondria under physiological conditions, but the damage is not enough to disturb mitochondrial homeostasis. Under external stimulation, ROS generation increased, and excessive accumulation of ROS caused mitochondrial homeostasis imbalance and led to mitochondrial damage, which resulted in mitophagy. To verify whether MnCl_2_ induces mitophagy in SH-SY5Y cells through ROS, we first assessed the level of ROS in SH-SY5Y cells after MnCl_2_ treatment. The results showed that, compared with the control treatment, treatment with increasing MnCl_2_ concentrations increased the levels of ROS in SH-SY5Y cells (Fig. 3a). In addition, we used the antioxidant N-acetyl cysteine (NAC) to determine whether MnCl_2_ induced ROS generation in SH-SY5Y cells. The results showed that NAC could reduce the production of ROS after MnCl_2_ treatment (Fig. 3b). The above data indicated that MnCl_2_ can promote ROS generation in SH-SY5Y cells. Therefore, we further explored whether excessive ROS production participates in MnCl_2_-mediated mitophagy in SH-SY5Y cells. The results showed that NAC can reduce the expression of the mitophagy receptor protein BNIP3, reduce the conversion of LC3-I to LC3-II and increase the expression of the mitochondrial marker protein TOMM20 (Fig. 3c). These data indicated that ROS participate in MnCl_2_-mediated mitophagy in SH-SY5Y cells. In addition, NAC also reduced the formation of mitochondrial autophagosomes (Fig. 3d). However, the autophagy inhibitor 3-MA did not reduce ROS generation (Fig. 3e). The above data further confirmed that ROS are involved in MnCl_2_-mediated mitophagy in SH-SY5Y cells.

### BNIP3 mediated MnCl_2_-induced mitophagy in SH-SY5Y cells

**Fig. 4:**
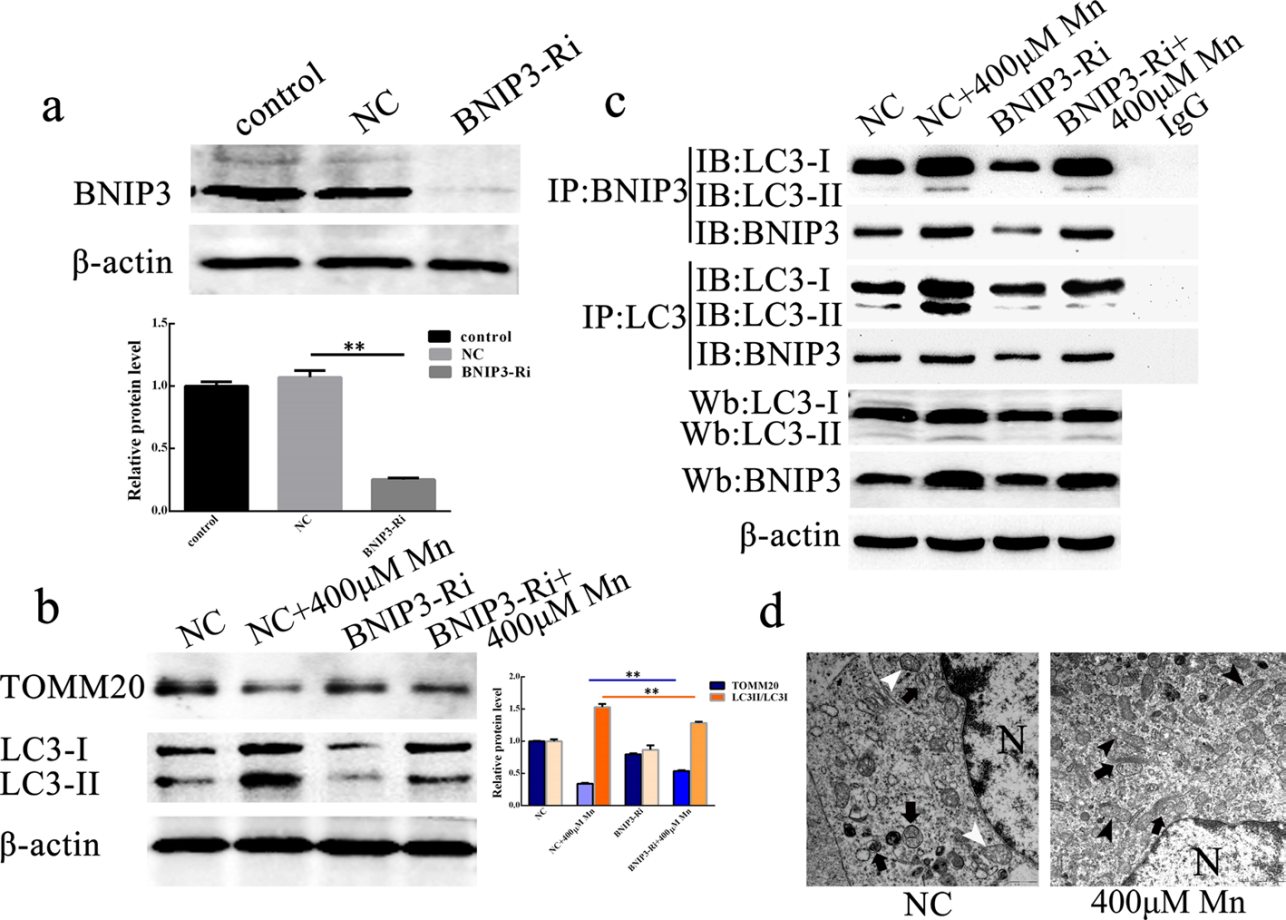
BNIP3 mediated MnCl_2_-induced mitophagy.  **a** BNIP3 levels in cells transfected with or without BNIP3 shRNA lentiviral vector were verified by western blot. β-Actin as internal reference. *P < 0.05, **P < 0.01. C, control treatment. NC, cells not transfected with lentivirus carrying interference sequences (also referred to as negative control). BNIP3-Ri, BNIP3-shRNA-transfected cells. **b** After treatment with 400 µM MnCl_2_, TOMM20 and LC3 protein levels were measured by western blot. β-actin served as the internal reference. Compared with NC, *P < 0.05, **P < 0.01. NC, negative control treatment. NC + Mn, 400 µM MnCl_2_ treated, not transfected with lentivirus interference sequence at 24 h. BNIP3-Ri, BNIP3-shRNA-transfected cells. BNIP3-Ri + Mn, 400 µM MnCl_2_-treated, BNIP3 shRNA-transfected cells at 24 h. **c** Co-immunoprecipitation result showed mitophagy receptor protein BNIP3 and autophagy marker protein LC3 bound to each other. MnCl_2_ promotes their binding, whereas BNIP3 shRNA transfection weakens this mechanism mediated by MnCl_2_. NC, negative control treatment. NC + Mn, cells treated with 400 µM MnCl_2_ but not transfected with lentivirus carrying interference sequences at 24 h. BNIP3-Ri, BNIP3 shRNA-transfected cells. BNIP3-Ri + Mn, 400 µM MnCl_2_-treated, BNIP3 shRNA-transfected cells at 24 h. **d** Mitophagy bodies and mitochondria in cells with normal or low BNIP3 expression were observed by TEM. N, nucleus. White arrow, expanded, condensed or dissolved, mitochondria aggregates. Black arrowhead (  ), relatively normal mitochondrial morphology. Black arrow (  ), mitophagy bodies

To explore whether BNIP3 mediated mitophagy, we used BNIP3 shRNA lentiviral vectors to interfere with BNIP3 expression. The results showed that the expression of the mitophagy receptor protein BNIP3 in the negative control was not significantly changed compared with the control but was significantly decreased in BNIP3-shRNA-transfected cells compared with the negative control, suggesting that the BNIP3 shRNA lentiviral vector could significantly reduce the expression of BNIP3 in SH-SY5Y cells, and the noninterfering sequence lentiviral vector had no significant effect on the expression of BNIP3 in SH-SY5Y cells (Fig. 4a). The western blot results showed that, compared with the negative control, 400 µM MnCl_2_ could increase the conversion of LC3-I to LC3-II in cells transfected with the noninterfering sequence lentiviral vector and BNIP3 shRNA for 24 h, but the increase in the former was obvious. MnCl_2_ (400 µM) also decreased the expression of the mitochondrial marker protein TOMM20, but the protein decrease in the BNIP3-shRNA-transfected cells was smaller than that in the noninterfering sequence lentivirus-transfected cells (Fig. 4b), suggesting that interference with BNIP3 could reduce MnCl_2_-induced mitophagy in SH-SY5Y cells. To further demonstrate that BNIP3 mediated MnCl_2_-induced mitophagy in SH-SY5Y cells, we verified whether BNIP3 regulated mitophagy progression by binding to the autophagy marker protein LC3 through coimmunoprecipitation. The results showed that the anti-BNIP3 antibody specifically captured and pulled down anti-LC3 antibody, and the anti-LC3 antibody specifically captured and pulled down the anti-BNIP3 antibody. These results suggested that BNIP3 and LC3 in SH-SY5Y cells could interact with each other effectively. Furthermore, it was demonstrated that MnCl_2_ could increase their binding, and decreased expression of BNIP3 could reduce their binding (Fig. 4c). In addition, transmission electron microscopy results showed that, after treatment with 400 µM MnCl_2_ for 24 h, the number of mitochondrial autophagosomes in the BNIP3-shRNA-transfected cells was lower than that in the noninterfering sequence lentivirus-transfected cells (Fig. 4d). Taken together, these results show that BNIP3 mediates MnCl_2_-induced mitophagy in SH-SY5Y cells.

### Silencing BNIP3 inhibited MnCl_2_-induced ROS generation and neurotoxicity

**Fig. 5:**
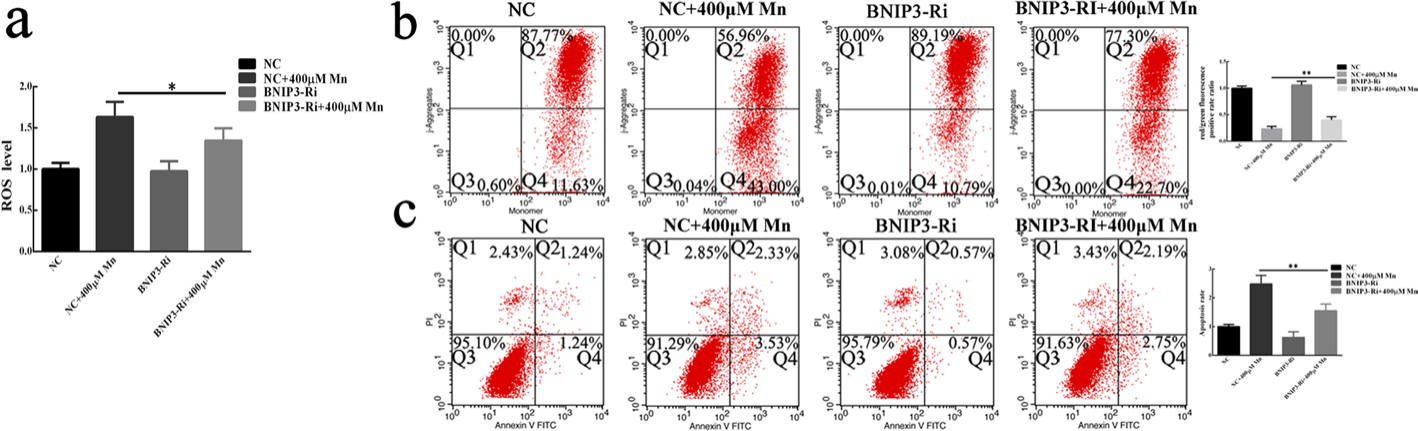
BNIP3 mediated MnCl_2_-induced ROS generation and neurotoxicity. **a**, **b**, **c** After treatment with or without 400 µM MnCl_2_, the levels of ROS (**a**), the mitochondrial membrane potential (**b**), and cell apoptosis (**c**) were assessed in cells with or without BNIP3 shRNA lentivirus. NC, negative control treatment. NC + Mn, cells treated with 400 µM MnCl_2_ but not treated with lentivirus carrying interference sequences at 24 h. BNIP3-Ri, BNIP3 shRNA-transfected cells. BNIP3-Ri + Mn, cells treated with 400 µM MnCl_2_ and transfected with BNIP3 shRNA at 24 h

Previous data indicated that BNIP3 knockdown could effectively reverse MnCl_2_-induced mitophagy in SH-SY5Y cells. Therefore, we further explored the role of BNIP3 in MnCl_2_-induced ROS generation and neurotoxicity in SH-SY5Y cells. Excitingly, compared with the negative control, the ROS generation in the noninterfering sequence lentivirus-transfected cells and the BNIP3-shRNA-transfected cells increased significantly after treatment with 400 µM MnCl_2_ for 24 h, but the former increased significantly compared with the latter (Fig. 5a). Furthermore, the mitochondrial membrane potential of both the noninterfering sequence lentivirus-transfected cells and the BNIP3-shRNA-transfected cells was significantly lost after 400 µM MnCl_2_ treatment for 24 h compared with the negative control treatment, but the mitochondrial membrane potential loss was smaller in the BNIP3-shRNA-transfected cells (Fig. 5b). The apoptosis results showed that the apoptosis rate of both the noninterfering sequence lentivirus-transfected cells and the BNIP3-shRNA-transfected cells was significantly higher after treatment with 400 µM MnCl_2_ for 24 h compared with the negative control treatment, but the apoptosis rate was lower in the BNIP3-shRNA-transfected cells (Fig. 5c). These results suggest that BNIP3 shRNA could inhibit MnCl_2_-induced ROS generation and mitochondrial membrane potential loss, leading to dopaminergic neuron toxicity. Taken together, these data demonstrate that BNIP3 mediates MnCl_2_-induced mitophagy and neurotoxicity in SH-SY5Y cells through oxidative stress.

## Discussion

The present study revealed for the first time that the mitophagy receptor protein BNIP3 mediated MnCl_2_-induced mitophagy in SH-SY5Y cells through ROS. This function may be one of the mechanisms responsible for MnCl_2_-induced neurotoxicity, suggesting that targeting BNIP3 is a potential strategy for the treatment of manganism and parkinsonism.

Manganese is widely distributed in the Earth’s crust, and it is one of the essential trace elements in the human body. Although manganese is associated with many biological functions, excessive exposure to manganese can cause damage to many organs and lead to manganism, resulting in neurodegenerative diseases and other related diseases. However, the molecular mechanism of this pathogenesis is unclear. Mitochondria play a key role in mammalian cells, especially in mitotic cells, such as neurons. Mitochondria have essential roles in energy balance, signal transduction and cellular metabolism [27–29]. Therefore, mitochondrial dysfunction can cause a variety of diseases [30–32], especially neurodegenerative diseases. Within a certain range of damaging stimuli, mitochondrial quality control can reverse or reduce neuronal death by promoting the formation of new mitochondria and removing damaged mitochondria [33]. In the absence of appropriate mitochondrial quality control, mitochondrial dysfunction increases and induces excessive mitophagy, leading to neuronal death [34]. Until now, the cause of dopaminergic neuron death remains unclear. Aging and genetic mutations may serve as potential pathogenic factors. In recent years, the environmental factors leading to the occurrence of parkinsonism have been receiving increasing attention from scientists. As early as 1837, movement disorders caused by occupational exposure to manganese led to a preliminary understanding of manganism and manganism-related parkinsonism. Although researchers have long been concerned about the association between manganese and manganism (parkinsonism) [35, 36], their shared regulatory mechanisms are still poorly understood. Therefore, further exploration of the mechanisms underlying manganese overexposure-mediated cellular damage will hopefully provide new clues for the treatment of manganism and parkinsonism.

BNIP3, Bcl2 interacting protein 3, is localized on the mitochondrial outer membrane, and BNIP3 has a special LIR motif structure that can bind to autophagy marker proteins to regulate mitophagy [37]. BNIP3 has been confirmed to mediate mitophagy under hypoxic conditions and other external stimuli as a mitophagy receptor protein [38]. However, the role of BNIP3-mediated mitophagy in manganese stimulation has rarely been reported. In our study, we found that MnCl_2_ could induce mitophagy in SH-SY5Y cells, thereby promoting the generation of ROS and triggering the apoptosis of SH-SY5Y cells in a dose-dependent manner. The obvious neurotoxic effect of Mn highlights the importance of clarifying its mechanism of action. Here, for the first time, we demonstrated that BNIP3 could mediate MnCl_2_-induced mitophagy through ROS in SH-SY5Y cells via serving as a mitophagy receptor protein. It has been suggested that BNIP3 promotes apoptosis by antagonizing the anti-apoptotic effect of E1B19kDa and Bcl2 through the BH3 domain [39]. This study also confirmed that BNIP3 mediated MnCl_2_-induced neurotoxicity in SH-SY5Y cells. The above data suggest that BNIP3 mediates manganese-induced mitophagy and leads to neurotoxicity through oxidative stress in dopaminergic neurons. However, there are still some limitations in the present work. For example, the specific regulatory mechanism of BNIP3-mediated mitophagy and neurotoxicity needs further study. In addition, although it is well known that MnCl_2_ can lead to manganism and parkinsonism by damaging mitochondria, little is known about the use of drugs to prevent mitochondrial damage or protect mitochondria. Whether the reversal of mitochondrial damage can control the development of diseases is rarely reported and still needs further exploration. Moreover, in our study, 3-MA failed to reverse the elevated ROS level in Mn-challenged SH-SY5Y cells. There are some possible reasons for the inability of 3-MA to reduce ROS generation. For example, 3-MA was reported to have off-target effect besides inhibiting autophagy [40]. Alternatively, a more complicated mechanism exists in Mn-induced ROS generation in SH-SY5Y cells, which needs further clarification.

In conclusion, BNIP3 contributes to manganese-induced mitophagy and neurotoxicity in dopaminergic SH-SY5Y cells through modulating ROS production.

## Data Availability

All data generated or analyzed during this study are included in this published article.

## Abbreviations

BNIP3: BCL2-interacting protein 3
ROS: Reactive oxygen species
LC3: Microtubule-associated protein 1 light chain 3 alpha
TOMM20: Translocase of outer mitochondrial membrane 20
3-MA: 3-methyladenine
MnCl_2_: Manganese(II) chloride
LIR: LC3-interacting region
shRNA: Short hairpin RNA

## References

[1] Hirsch L, Jette N, Frolkis A, Steeves T, Pringsheim T (2016). The incidence of Parkinson’s disease: a systematic review and meta-analysis. Neuroepidemiology.

[2] Rocca WA (2018). The burden of Parkinson’s disease: a worldwide perspective. Lancet Neurol.

[3] Ball N, Teo WP, Chandra S, Chapman J (2019). Parkinson’s disease and the environment. Front Neurol.

[4] Nandipati S, Litvan I (2016). Environmental exposures and Parkinson’s disease. Int J Environ Res Public Health.

[5] Hatcher JM, Pennell KD, Miller GW (2008). Parkinson’s disease and pesticides: a toxicological perspective. Trends Pharmacol Sci.

[6] Caudle WM, Guillot TS, Lazo CR, Miller GW (2012). Industrial toxicants and Parkinson’s disease. Neurotoxicology.

[7] Garza-Lombó C, Posadas Y, Quintanar L, Gonsebatt ME, Franco R (2018). Neurotoxicity linked to dysfunctional metal ion homeostasis and xenobiotic metal exposure: redox signaling and oxidative stress. Antioxid Redox Signal.

[8] Schroeder HA, Balassa JJ, Tipton IH (1966). Essential trace metals in man: manganese. A study in homeostasis. J Chronic Dis.

[9] Tuschl K, Mills PB, Clayton PT (2013). Manganese and the brain. Int Rev Neurobiol.

[10] Martinez-Finley EJ, Gavin CE, Aschner M, Gunter TE (2013). Manganese neurotoxicity and the role of reactive oxygen species. Free Radic Biol Med.

[11] Wan C, Ma X, Shi S, Zhao J, Nie X, Han J (2014). Pivotal roles of p53 transcription-dependent and -independent pathways in manganese-induced mitochondrial dysfunction and neuronal apoptosis. Toxicol Appl Pharmacol.

[12] Gorojod RM, Alaimo A, Porte Alcon S, Pomilio C, Saravia F, Kotler ML (2015). The autophagic- lysosomal pathway determines the fate of glial cells under manganese- induced oxidative stress conditions. Free Radic Biol Med.

[13] Zhang J, Cao R, Cai T, Aschner M, Zhao F, Yao T (2013). The role of autophagy dysregulation in manganese-induced dopaminergic neurodegeneration. Neurotox Res.

[14] Guilarte TR (2010). Manganese and Parkinson’s disease: a critical review and new findings. Environ Health Perspect.

[15] Humphrey DM, Parsons RB, Ludlow ZN, Riemensperger T, Esposito G, Verstreken P (2012). Alternative oxidase rescues mitochondria-mediated dopaminergic cell loss in Drosophila. Hum Mol Genet.

[16] Baker MJ, Tatsuta T, Langer T (2011). Quality control of mitochondrial proteostasis. Cold Spring Harb Perspect Biol.

[17] Sarraf SA, Raman M, Guarani-Pereira V, Sowa ME, Huttlin EL, Gygi SP (2013). Landscape of the PARKIN-dependent ubiquitylome in response to mitochondrial depolarization. Nature.

[18] Liu WJ, Ye L, Huang WF, Guo LJ, Xu ZG, Wu HL (2016). p62 links the autophagy pathway and the ubiqutin-proteasome system upon ubiquitinated protein degradation. Cell Mol Biol Lett.

[19] Hanna RA, Quinsay MN, Orogo AM, Giang K, Rikka S, Gustafsson ÅB (2012). Microtubule-associated protein 1 light chain 3 (LC3) interacts with Bnip3 protein to selectively remove endoplasmic reticulum and mitochondria via autophagy. J Biol Chem.

[20] Lei Q, Tan J, Yi S, Wu N, Wang Y, Wu H (2018). Mitochonic acid 5 activates the MAPK-ERK-yap signaling pathways to protect mouse microglial BV-2 cells against TNFα-induced apoptosis via increased Bnip3-related mitophagy. Cell Mol Biol Lett.

[21] Kanki T (2010). Nix, a receptor protein for mitophagy in mammals. Autophagy.

[22] Liu L, Feng D, Chen G, Chen M, Zheng Q, Song P (2012). Mitochondrial outer-membrane protein FUNDC1 mediates hypoxia-induced mitophagy in mammalian cells. Nat Cell Biol.

[23] Nakamura Y, Kitamura N, Shinogi D, Yoshida M, Goda O, Murai R (2012). BNIP3 and NIX mediate Mieap-induced accumulation of lysosomal proteins within mitochondria. PLoS One.

[24] Šprung M, Dikic I, Novak I (2018). Flow cytometer monitoring of Bnip3- and Bnip3L/Nix-dependent mitophagy. Methods Mol Biol.

[25] Ghavami S, Eshragi M, Ande SR, Chazin WJ, Klonisch T, Halayko AJ (2010). S100A8/A9 induces autophagy and apoptosis via ROS-mediated cross-talk between mitochondria and lysosomes that involves BNIP3. Cell Res.

[26] Prabhakaran K, Chapman GD, Gunasekar PG (2009). BNIP3 up-regulation and mitochondrial dysfunction in manganese-induced neurotoxicity. Neurotoxicology.

[27] Sena LA, Chandel NS (2012). Physiological roles of mitochondrial reactive oxygen species. Mol Cell.

[28] Schumacker PT, Gillespie MN, Nakahira K, Choi AM, Crouser ED, Piantadosi CA (2014). Mitochondria in lung biology and pathology: more than just a powerhouse. Am J Physiol Lung Cell Mol Physiol.

[29] Fazal L, Laudette M, Paula-Gomes S, Pons S, Conte C, Tortosa F (2017). Multifunctional mitochondrial epac1 controls myocardial cell death. Circ Res.

[30] Faizi M, Seydi E, Abarghuyi S, Salimi A, Nasoohi S, Pourahmad J (2016). A search for mitochondrial damage in Alzheimer’s disease using isolated rat brain mitochondria. Iran J Pharm Res.

[31] Krzysztoń-Russjan J (2016). Pathophysiology and molecular basis of selected metabolic abnormalities in Huntington’s disease. Postepy Hig Med Dosw (Online).

[32] Zhao D, Zheng H, Greasley A, Ling F, Zhou Q, Wang B (2020). The role of miR-711 in cardiac cells in response to oxidative stress and its biogenesis: a study on H9C2 cells. Cell Mol Biol Lett.

[33] East DA, Campanella M (2016). Mitophagy and the therapeutic clearance of damaged mitochondria for neuroprotection. Int J Biochem Cell Biol.

[34] Dukes AA, Bai Q, Van Laar VS, Zhou Y, Ilin V, David CN (2016). Live imaging of mitochondrial dynamics in CNS dopaminergic neurons in vivo demonstrates early reversal of mitochondrial transport following MPP(+) exposure. Neurobiol Dis.

[35] Park RM (2013). Neurobehavioral deficits and parkinsonism in occupations with manganese exposure: a review of methodological issues in the epidemiological literature. Saf Health Work.

[36] Bouabid S, Tinakoua A, Lakhdar-Ghazal N, Benazzouz A (2016). Manganese neurotoxicity: behavioral disorders associated with dysfunctions in the basal ganglia and neurochemical transmission. J Neurochem.

[37] Shi RY, Zhu SH, Li V, Gibson SB, Xu XS, Kong JM (2014). BNIP3 interacting with LC3 triggers excessive mitophagy in delayed neuronal death in stroke. CNS Neurosci Ther.

[38] Zhu Y, Massen S, Terenzio M, Lang V, Chen-Lindner S, Eils R (2013). Modulation of serines 17 and 24 in the LC3-interacting region of Bnip3 determines pro-survival mitophagy versus apoptosis. J Biol Chem.

[39] Walls KC, Ghosh AP, Ballestas ME, Klocke BJ, Roth KA (2009). bcl-2/Adenovirus E1B 19-kd interacting protein 3 (BNIP3) regulates hypoxia-induced neural precursor cell death. J Neuropathol Exp Neurol.

[40] Sefton EC, Qiang W, Serna V, Kurita T, Wei JJ, Chakravarti D (2013). MK-2206, an AKT inhibitor, promotes caspase-independent cell death and inhibits leiomyoma growth. Endocrinology.

